# Tuning the Diradical Character of Indolocarbazoles: Impact of Structural Isomerism and Substitution Position

**DOI:** 10.1021/acs.jpclett.2c01325

**Published:** 2022-06-23

**Authors:** Irene Badía-Domínguez, Sofia Canola, Víctor Hernández Jolín, Juan T. López Navarrete, Juan C. Sancho-García, Fabrizia Negri, M. Carmen Ruiz Delgado

**Affiliations:** † Department of Physical Chemistry, University of Málaga, Campus de Teatinos s/n, 29071 Málaga, Spain; ‡ Department of Chemistry “Giacomo Ciamician”, University of Bologna, 40126 Bologna, Italy; § Department of Physical Chemistry, University of Alicante, 03080 Alicante, Spain; ∥ INSTM, UdR Bologna, 40126 Bologna, Italy

## Abstract

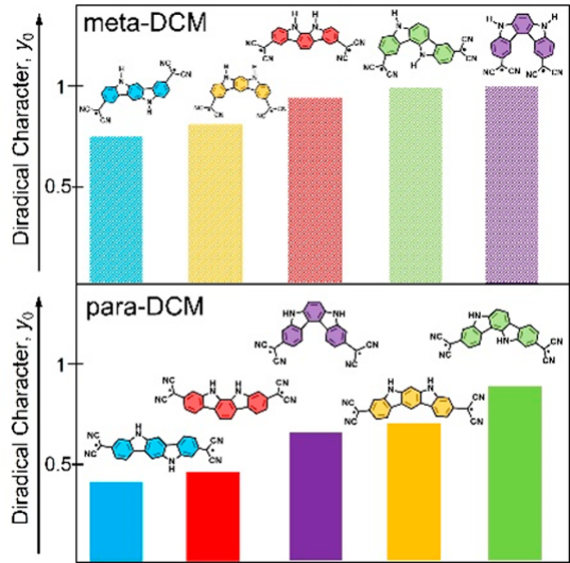

In this study, a set of 10 positional indolocarbazole (ICz) isomers substituted with dicyanomethylene groups connected via *para* or *meta* positions are computationally investigated with the aim of exploring the efficiency of structural isomerism and substitution position in controlling their optical and electronic properties. Unrestricted density functional theory (DFT), a spin-flip time-dependent DFT approach, and the multireference CASSCF/NEVPT2 method have been applied to correlate the diradical character with the energetic trends (i.e., singlet–triplet energy gaps). In addition, the nucleus-independent chemical shift together with ACID plots and Raman intensity calculations were used to strengthen the relationship between the diradical character and (anti)aromaticity. Our study reveals that the substitution pattern and structural isomerism represent a very effective way to tune the diradical properties in ICz-based systems with *meta*-substituted systems with a V-shaped structure displaying the largest diradical character. Thus, this work contributes to the elucidation of the challenging chemical reactivity and physical properties of diradicaloid systems, guiding experimental chemists to produce new molecules with desirable properties.

Diradical systems present two unpaired electrons localized at two different regions with a non-negligible interaction. The coupling interaction between the radical centers determines (i) the open-shell (OS) singlet or triplet (T) ground state and (ii) the degree of diradical character of a molecule fluctuating from pure diradical (*y*_0_ = 1) to closed-shell (CS) electronic structure (*y*_0_ = 0). Particularly interesting is the case of OS singlet diradical molecules, which feature a large number of relevant properties compared to CS systems, for instance, a small HOMO–LUMO gap (H–L gap), largely improved two-photon absorption (TPA) toward the near-infrared (near-IR) region, or a remarkable magnetic activity emerging from thermally populated triplet states.^1−3^ However, diradicals share a complicated electronic structure; one configuration dominates the electronic wave function, and several possess equal or similar weights. In fact, although OS and CS notations for singlet states are frequently used in the literature, some alternative terms also exist (i.e., “disjoint” or “joint” diradicals based on a delocalized to localized orbital transformation that interchanges CS and OS descriptions), as has been shown in a recent review.^4^

In the past several decades, the chemistry of diradical systems, mainly the preparation of stable long-lived OS molecules, has been greatly developed to exploit these systems in many promising applications in the fields of organic electronics and spintronics and to design nonlinear optical devices.^5−12^ Quantum chemical (QC) investigations are essential for determining structure–property relationships for these molecules and for rationalizing the variations of electronic and geometrical properties as a function of the degree of OS singlet nature.^13−16^ Among others, the quantitative estimation of diradical character with QC approaches is remarkably relevant because it enables a deeper understanding of the nature of chemical bonds, thereby illuminating the best structure–property relationships in OS molecular systems. A widely used diradical character descriptor is the *y*_0_ parameter, defined as twice the weight of the doubly excited configuration,^17−20^ and it can be calculated via spin-unrestricted approaches.^21^ More recently, other descriptors have been proposed, such as the *N*^FOD^ parameter, based on finite-temperature density functional theory (FT-DFT) and measuring the appearance of “hot” or strongly correlated electrons.^22,23^

In the past few years, there has been more interest in the rationalization of how the extent of diradical character is influenced by structural changes introduced to stabilize (or destabilize) the diradical system. Because of the tremendous effort of organic chemists, more stable singlet diradical molecules with controllable amounts of diradical character have been synthesized and characterized.^24,25^

The tunability of the diradical character has therefore been studied for several different structural motifs: (i) the substitution position of lateral groups (indeed, different synthetic strategies have been considered with the aim of controlling the degree of diradical character as a function of the substitution pattern and different lateral substitution),^26−30^ (ii) chemical modification of π-bridges,^31,32^ (iii) elongation of the conjugated core, because extended π-systems involve greater OS singlet structure stability^33−42^ (for instance, it has been demonstrated that the extension on the bridge when going from monophenylene to biphenylene and triphenylene results in an increase in the level of diradical character because of an increase in aromaticity),^43−46^ and (iv) influence of isomerism on the chemical properties and, consequently, the diradical character.^47−50^

In recent works, the investigation of structural isomerism of carbon-based diradical systems has provided a reliable assessment of how the small geometric changes impact the properties of the system due to the alterations in the conjugation backbone.^49−52^ However, nitrogen-centered singlet diradicals have seldom been explored because of synthetic challenges.^53−55^ In fact, while different reports dealt with the investigation of indenofluorene-based diradicals,^40,48,56,57^ their nitrogen-centered indolocarbazole (ICz) analogues have been scarcely investigated. To the best of our knowledge, our recent work on the 3,9-dicyanomethylene-indolo[3,2-*b*]carbazole [named *p***-32b-ICz** in Figure 1 (see also Figure S1)] is the only study to date that has focused on ICz-based diradicaloids.^58^ Due to the large diradical character and interesting electronic properties found for *p***-32b-ICz**, here we extend our study to the influence of structural isomerism in ICz-based diradicals.

**Figure 1:**
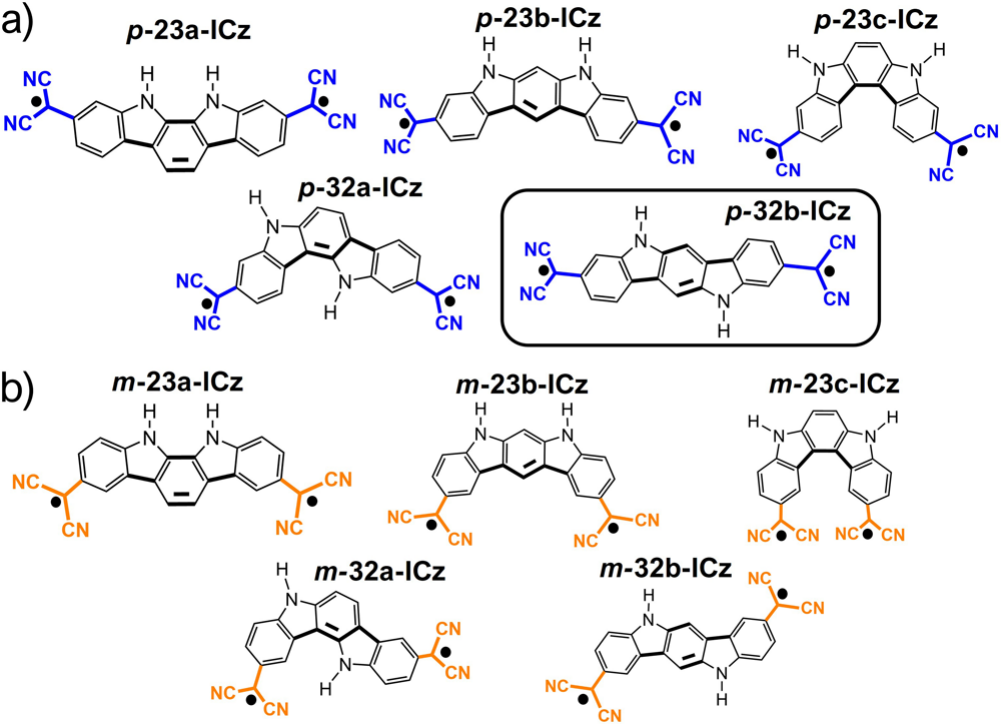
Chemical structures (diradical resonance structures) of the set of positional ICz isomers substituted with dicyanomethylene groups connected via (a) *para* or (b) *meta* positions. The already synthesized *p***-32b-ICz** system is shown in a box.

Indolo[3,2-*b*]carbazole is only one member of this family that also possesses four additional isomers with different phenyl linkages in *ortho* (**23c-ICz**), *meta* (**23b-ICz** and **32a-ICz**), and *para* (**23a-ICz** and **32b-ICz**) positions and different bridge structures (*anti* for **32a-ICz** and **32b-ICz** vs *syn* for **23a-ICz**, **23b-ICz**, and **23c-ICz**) (see Figure S1). Nevertheless, there has been limited discussion of the physical properties and chemical reactivity of these other four isomers.^57,59−63^ In addition, via insertion of dicyanomethylene (DCM) groups at different positions (*para* or *meta*), a library of 10 ICz isomers is obtained, which represents a good data set for understanding the consequences of the changes in topology for the diradical character. Herein, we then investigate a set of 10 ICz isomers substituted with DCM groups (Figure 1) to disclose how their chemical reactivity and physical properties are affected by structural isomerism and different substitution patterns. We believe that this study provides new design guidelines for better efficient functional diradical systems.

DFT calculations were performed to explore the electronic structure and the diradical stability of these ICz-based isomers by means of different physical parameters such as (i) the diradical character, (ii) the effective electron exchange interaction (*J*_ab_), (iii) the singlet–triplet energy gap (Δ*E*_S-T_), and (iv) the energy difference between OS singlet and CS singlet states (Δ*E*_OS-CS_). As shown in Table 1, the diradical character can be remarkably tuned upon positional isomerism (i.e., from *y*_0_ = 0.45 to *y*_0_ = 0.89 on going from *p***-23a-ICz** to *p***-32a-ICz**) and by varying the DCM substitution position (i.e., from *y*_0_ = 0.45 to *y*_0_ = 0.93 from *p***-23a-ICz** to *m***-23a-ICz**). The *J*_ab_ parameter, which reflects the overlap integral between the two nearly energetically degenerate molecular orbitals, gives positive *J*_ab_ values for the molecules displaying the largest diradical character (i.e., *p***-32a-ICz**, *m***-23a-ICz**, *m***-23c-ICz**, and *m***-32a-ICz**), indicating their triplet ground state. On the contrary, the molecules showing the lowest diradical character (i.e., *p***-23a-ICz**, *p***-23c-ICz**, and *p***-32b-ICz)** display the highest negative *J*_ab_ values within the series, that is, a moderate interaction between two spins. In addition, *N*^FOD^ values of >1.5 are found for the whole set of ICz systems; this indicates a highly pronounced diradical character, especially in the case of the DCM *meta*-substituted systems. Interestingly, a linear relationship is found between the *N*^FOD^ values and the theoretical *y*_0_ values (Figure S2). The density plot arising from the fractionally occupied orbital shows that the spatial distribution of the unpaired electrons is highly delocalized over the whole conjugated backbone in the DCM *para*-substituted ICz systems but in the *meta*-substituted analogues is more localized over the external indoles and adjacent DCM groups, although in all cases the largest contribution derived from the central carbon atoms of DCM groups (Figure S3).

**Table 1:** Diradical Indices *y*_0_ and *N*^FOD^ and Physical Properties for ICz-Based Systems at the UB3LYP/6-31G** Level

	⟨*S*^2^⟩_OS_	⟨*S*^2^⟩_T_	*N*^FOD^ (TPSS/def2-TZVP)	*y*_0_ (PUB3LYP)	*J*_ab_ (kcal mol^–1^)
*p***-23a-ICz**	0.96	2.04	1.83	0.45	–1.42
*p***-23b-ICz**	1.02	2.05	2.01	0.69	–0.04
*p***-23c-ICz**	1.02	2.05	2.05	0.66	–0.45
*p***-32a-ICz**	1.04	2.05	2.12	0.89	0.40
*p***-32b-ICz**	0.94	2.04	1.85	0.40	–1.86
*m***-23a-ICz**	1.04	2.04	2.15	0.93	0.02
*m***-23b-ICz**	1.03	2.04	2.01	0.79	–0.08
*m***-23c-ICz**	1.04	2.04	2.18	0.99	0.07
*m***-32a-ICz**	1.04	2.04	2.15	0.98	0.17
*m-***32b-ICz**	1.03	2.04	1.98	0.75	–0.27

Interestingly, it should be highlighted that all systems are characterized by planar backbone conformations except for **23c-ICz** isomers, which display twisted structures that are especially relevant upon substitution [i.e., with bay dihedral angles, θ, of 6° in **23c-ICz**, 10° in *p***-23c-ICz**, and 22° in *m***-23c-ICz** (see Figure S4)]. On the contrary, the V-shaped structure of *m***-23c-ICz** results in large dipole moments [∼15 D (see Figure S5 and Table S2)] in both singlet and triplet states, indicating efficient charge separation and a pronounced zwitterionic character. Thus, distortions from planarity and large dipole moments could involve remarkable modifications of the diradical character;^64,65^ in fact, the *m***-23c-ICz** isomer displays the largest diradical character within the series.

The reasons for ICz isomers having a large diradical character are further discussed from the point of view of Δ*E*_S-T_ and Δ*E*_OS-CS_. As Figure 2 shows, the negative values of Δ*E*_OS-CS_ reveal that all DCM-substituted systems are OS diradicals in the ground state, with this effect being more significant when the DCM groups are connected at the *meta* position. The molecules displaying the largest diradical character are also those showing the largest Δ*E*_OS-CS_ and the smallest Δ*E*_S-T_; note that small Δ*E*_S-T_ values are generally accompanied by weak coupling of the unpaired electrons and, thus, by large diradical character. Therefore, we are able to define a linear correlation between diradical character *y*_0_ and these two factors (Δ*E*_S-T_ and Δ*E*_OS-CS_) for the whole set of isomers, suggesting that changes in the substitution pattern and structural isomerism represent a very effective way to modulate the diradical properties. Interestingly, the library of ICz systems can be divided into two subgroups according to the terminal DCM substitution position: (i) the *para*-substituted systems that span a wider range of *y*_0_ values (∼0.4 and 0.9) and (ii) the *meta*-substituted homologues that display larger *y*_0_ values in a much narrower range (∼0.7 and 0.95).

**Figure 2:**
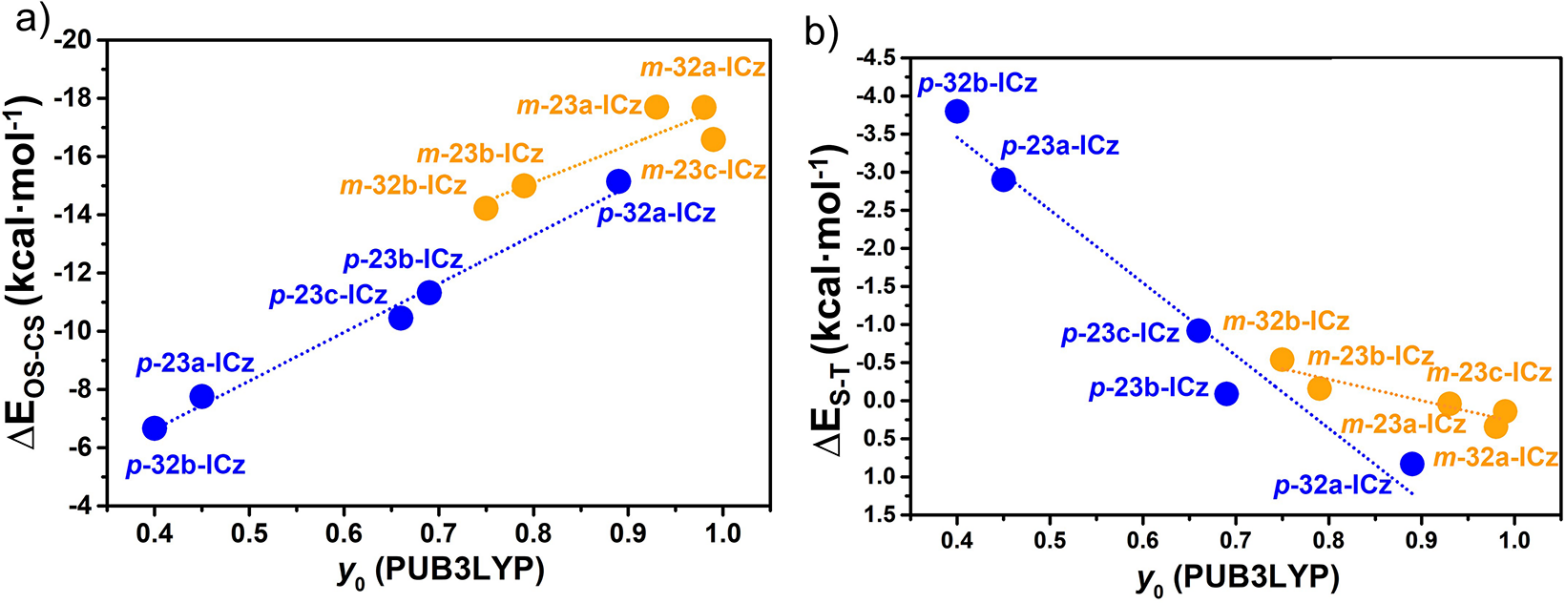
Correlation between diradical character *y*_0_ calculated at the projected UB3LYP/6-31G** level and (a) Δ*E*_OS-CS_ and (b) Δ*E*_S-T_ for DCM *para*-substituted (blue circles) and *meta*-substituted (orange circles) systems.

In addition to the unrestricted DFT methods, the spin-flip TD-DFT approach and the multireference CASSCF method followed by NEVPT2 corrections have been applied to validate the observed energetic trends. While CASSCF calculations^66^ can correctly describe the static electron correlation affecting electronic states with multiconfiguration character, naturally fixing the main failures of standard single-reference methods, spin-flip methods have been successfully applied to study diradicals^67−69^ combining low computational cost with results approaching multireference quality. Table 2 compares the singlet–triplet energy gaps (Δ*E*_S-T_) for the different methods (TD-DFT, the spin-flip (SF)-TD-DFT, and CASSCF/NEVPT2). CASSCF/NEVPT2 results are quite similar to those predicted for UDFT, while SF-TD-DFT values seem to be overestimated. Nevertheless, the three sets of Δ*E*_S-T_ data give similar trends within the series, confirming that our results based on the unrestricted DFT method should be trustworthy.

**Table 2:** Comparison between the Singlet–Triplet Energy Gap (Δ*E*_S-T_) Values Computed by TD-DFT, SF-TD-DFT, and CASSCF/NEVPT2 Methods and Diradical Indices *y*_0_ for ICz-Based Systems

	*y*_0_ (PUB3LYP)	Δ*E*_S-T_ (UB3LYP) (kcal mol^–1^)	Δ*E*_S-T_ (SF-TDB3LYP) (kcal mol^–1^)	Δ*E*_S-T_ [CASSCF(10,10)/NEVPT2] (kcal mol^–1^)
*p***-23a-ICz**	0.45	–2.90	–5.05	–2.08
*p***-23b-ICz**	0.69	–0.09	–1.41	–0.18
*p***-23c-ICz**	0.66	–0.92	–1.63	–0.23
*p***-32a-ICz**	0.89	0.83	0.47	0.18
*p***-32b-ICz**	0.40	–3.80	–6.26	–3.00
*m***-23a-ICz**	0.93	0.04	0.01	–0.12
*m***-23b-ICz**	0.79	–0.16	–0.68	–0.12
*m***-23c-ICz**	0.99	0.14	0.14	0.00
*m***-32a-ICz**	0.98	0.34	0.65	0.07
*m-***32b-ICz**	0.75	–0.54	–1.08	–0.16

Interestingly, a distinctive feature of OS singlet diradical systems is the presence of a low-lying double exciton state dominated by the H,H → L,L excitation.^70−74^ Recently, several QC investigations have proven that this state can become the lowest singlet excited state for compounds with large diradical character, a feature that has been experimentally supported for several conjugated diradicals and may influence their photoresponse properties. The excitation energy of the double exciton state has been calculated for the 10 isomers by using two different approaches, extending the standard time-dependent DFT treatment (TD-DFT): the spin-flip (SF-)TD-DFT method and calculations based on unrestricted broken symmetry antiparallel-spin reference configuration (TD-UDFT) (Figure S6). The results show that SF-TD-DFT calculations generally underestimate the excitation energy of the double exciton state with this effect being more pronounced in compounds with larger diradical character. This is in line with previous investigations in which it has been shown that for very large diradical character values the TD-UDFT calculations provide a reliable excitation energy prediction for both the double exciton state dominated by the H,H → L,L excitation and the single exciton state dominated by the H → L excitation.^71^

The quality of predictions can be appreciated in Figure 3 where the experimental and computed spectra of *p***-32b-ICz** are compared. As shown in Figure 3a, the experimental electronic spectrum of *p***-32b-ICz** shows a strong absorption at 1.76 eV together with two weak shoulders at lower energies (1.58 and 1.44 eV). On the basis of the computed results, the first intense band is readily assigned to the H → L transition (determined from the OS geometry at 1.72 eV) while the low-energy shoulders are assigned to the double exciton state (two low-lying excited states computed at the TD-UB3LYP level at 1.28 and 1.33 eV are dominated by the H,H → L,L excitation).

**Figure 3:**
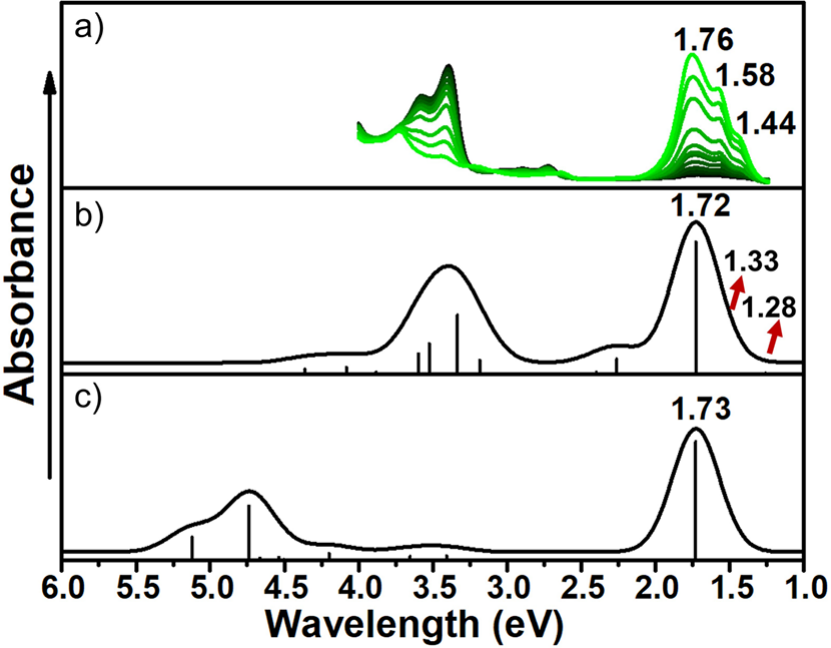
(a) Ultraviolet–visible–NIR absorption spectra of *p***-32b-ICz** in *o*-dichlorobenzene upon heating from 300 to 410 K. (b) Simulated TD-UDFT electronic absorption spectra (UB3LYP/6-31G**) of the *p***-32b-ICz** OS structure. The red arrows show the theoretical values of the one-photon forbidden excited states dominated by the H,H → L,L excitation. (c) Simulated TD-DFT electronic absorption spectra (B3LYP/6-31G**) of the *p***-32b-ICz** CS structure.

Because the low-lying strong absorption band is due to the H → L transition, it is interesting to compare the differences in the H–L gap when comparing the CS and OS structures for the entire compound series and to determine its dependence on the diradical character. As shown in Figure 4, the OS structures present approximately constant H-L gap values that can be rationalized by their slight structural changes, as we will describe below. In contrast, the CS structures display a remarkable H–L gap decrease for increased diradical character that is more significant in the case of laterally *para*-substituted compounds, suggesting in this case relevant geometrical changes between the CS and OS states. Indeed, this is confirmed by the computed structural changes taking place when going from quinoid CS to the more aromatic OS structure, which are remarkably larger for *para* derivatives (Figure S8) than for *meta* derivatives (Figure S9). It should be noted that for two of the *para*-substituted compounds displaying the largest CS to OS H–L gap change (*p***-23b-ICz** and *p***-32a-ICz)**, the CS form cannot show an exact quinoid structure because of the interruption of the linear conjugation caused by the phenyl linkages in the *meta* position (Figures S10 and S11). To gain a deeper understanding of these alterations in the conjugated backbone, the degree of aromatization with different parameters will be explored in the next paragraph.

**Figure 4:**
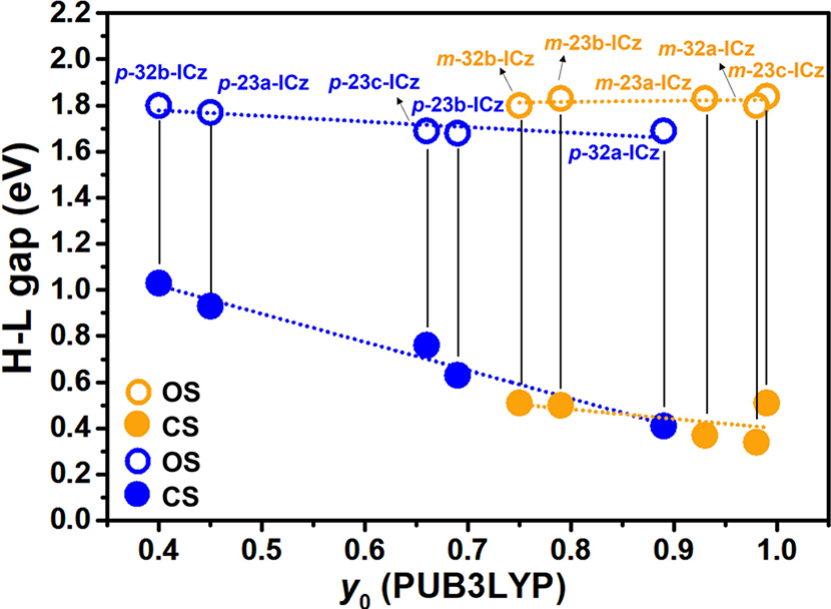
Computed projected diradical character at the UB3LYP/6-31G** level of theory vs the H–L gap of both OS (empty circles) and CS (filled circles) structures for *para* (blue circles) and *meta* (orange circles) positions of the DCM groups. Vertical bars indicate the compound to which computed data correspond.

We now examine how the aromaticity and antiaromaticity of ICz-based systems can be controlled by structural isomerism and substitution position. Toward this end, the ring currents were examined computationally with the nucleus-independent chemical shift NICS(0),^75^ the NICS-XY scan,^76−78^ and the anisotropy of the induced current density (ACID) method.^79,80^ Negative NICS values indicate aromaticity (diatropic ring current), while positive values indicate antiaromaticity (paratropic ring current); thus, a reduction in the diatropic ring current is associated with a partial gain in quinoid character.^81,82^Figure 5 shows the NICS-XY scans^76−78^ of three different isomers (*p***-32b-ICz**, *p***-23b-ICz**, and *p***-32a-ICz**) with distinct diradical character. Several points should be highlighted. (i) In comparison with the unsubstituted isomers, the insertion of DCM groups results in less negative NICS-XY values and thus decreased aromaticity. (ii) Slight differences are predicted in *p***-32b-ICz** (*y*_0_ = 0.40) when going from the external rings to the central core (with negative and positive NICS values close to zero), while slightly larger differences are found for *p***-23b-ICz** (*y*_0_ = 0.69), thus leading to less conjugation between the external and central parts of the ICz core in accordance with the greater diradical character. (iii) A further increase in diradical character results in more intense changes in the central part of the system. For instance, the central benzene ring of *p***-32a-ICz** (*y*_0_ = 0.89) displays an increase in the paratropic ring current translating into an antiaromatic ring while the external benzene rings retain the aromatic character.

**Figure 5:**
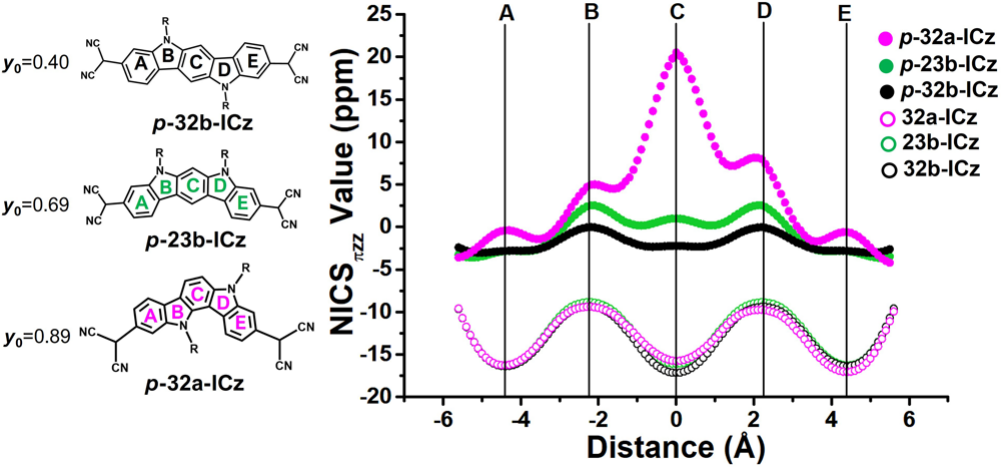
NICS_πz_-XY scan values (B3LYP/6-311+G* level) and chemical structures of *p***-32b-ICz** (black circles), *p***-23b-ICz** (green circles), and *p***-32a-ICz** (pink circles). The NICS_πz_-XY scan values of unsubstituted isomer analogues (**32b-ICz**, **23b-ICz**, and **32a-ICz**) are also shown for comparison.

Interestingly, the ACID plots shown in Figure 6 endorse the NICS values, showing that an increase in diradical character translates in the presence of antiaromatic rings in the central core, which indicates less π-delocalization of the conjugated backbone. On the contrary, much larger differences in the aromatic/antiaromatic character within the conjugated backbone are found in the peripheral *meta*-substituted systems when compared with the *para*-substituted analogues (Figures S12 and S13). This can be ascribed to a decrease in the level of π-conjugation upon DCM substitution at the *meta* position because of the interruption of the linear π-system, which in turn promotes diradical character.

**Figure 6:**
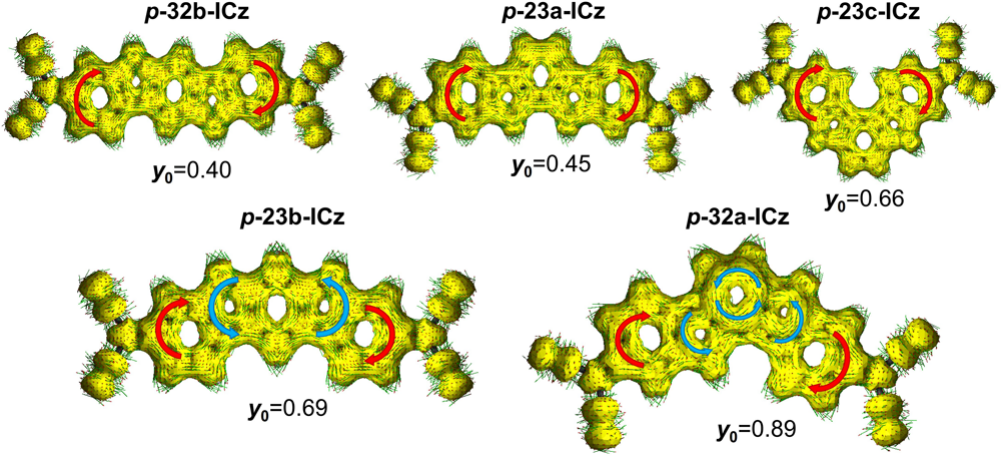
ACID plots of the induced ring current at an isosurface value of 0.03 of the *para*-substituted ICz under study. The red arrows represent a diatropic (clockwise) ring current, and the blue arrows correspond to a paratropic (counterclockwise) ring current. The computed projected diradical character at the UB3LYP/6-31G** level is also shown.

Finally, the NICS(0) values calculated for all ICz isomers (Figure S14) demonstrate that a large increase in aromatic character is found when going from the CS to OS state, with this effect being more intense in the peripheral *meta*-substituted systems, thus favoring the diradical character and further demonstrating that a large diradical character is driven by the recovery of aromaticity. In addition, Raman spectroscopy is used as a complementary technique to explore the changes in the molecular structures of these diradical systems. A frequency upshift of the bands associated with the ν(CC) stretching vibrations of the ICz backbone is detected when going from the peripheral *para*- to *meta*-substituted isomers in their OS states (Figures S15–S19). This is in consonance with the structural differences mentioned, pointing to an increased degree of aromatization in the central conjugated backbone in the *meta*-substituted systems, which would in turn promote diradical character.

In this study, we theoretically investigate how structural isomerism and substitution position affect the optical and electronic properties of DCM-substituted indolocarbazoles. The library of 10 isomers can be divided into two subgroups as a function of the DCM substitution position: the *para*-substituted systems with diradical character (*y*_0_) values spanning a wide range (∼0.4–0.9) and the *meta*-substituted homologues displaying much larger *y*_0_ values in a narrower range (∼0.7–0.95). Large differences in aromatic and/or antiaromatic character within the conjugated backbone are found in the DCM *meta*-substituted systems, which results in a decreased level of π-conjugation thus promoting greater diradical character. The correlation predicted at the UDFT level between the diradical character and the singlet–triplet energy gap (Δ*E*_S-T_) values has been also validated with electronic structure calculations using the multireference CASSCF/NEVPT2 method and the SF-TD-DFT approach. In addition, the presence of double exciton states dominated by the H,H → L,L excitation has been successfully provided with a nice agreement found with the experimental absorption spectra of *p***-32b-ICz**. Overall, these results demonstrate how small geometric changes impact the properties of DCM *meta*-substituted systems with a V-shaped structure and large dipole moments displaying the greatest diradical character within the series. Thus, this study provides new molecular design strategies toward the development of diradicaloid molecules with potential use in different material applications.

## Supporting Information Available

Computational details; diradical indices *y*_0_, *N*^FOD^ values, and physical properties of ICz-based systems at the UM06-2X/6-31G** level; isocontour plots of the FOD density; calculated dipole moments; calculated excitation energies of the one-photon forbidden (H,H → L,L) state versus the computed projected diradical character; simulated electronic absorption spectra at the UM06-2X/6-31G** level of theory; computed bond lengths for the optimized CS and OS structures; equilibrium between the diradical character OS and the quinoid CS structures; NICS_πzz_-XY scan values and ACID plots for the laterally *meta*-substituted systems and NICS(0) values for ICz-based systems; and theoretical Raman spectra (PDF)
