# Supplementary material for: Tuning the Diradical Character of Indolocarbazoles: Impact of Structural Isomerism and Substitution Position
Source: J Phys Chem Lett. 2022 Jun 23;13(26):6003–10. doi: 10.1021/acs.jpclett.2c01325 (PMC9272443; doi:10.1021/acs.jpclett.2c01325)
Supplement: Supplementary file 1 — jz2c01325_si_001.pdf [file jz2c01325_si_001.pdf]

SUPPORTING INFORMATION

Tuning the Diradical Character of Indolocarbazoles: Impact of Structural Isomerism and Substitution Position

Irene Badía-Domínguez,[a] Sofia Canola,[b] Víctor Hernández Jolín,[a] Juan T. López Navarrete,[a] J. Carlos Sancho-García,[c] Fabrizia Negri,[b,d*] M. Carmen Ruiz Delgado[a*]

a Department of Physical Chemistry, University of Málaga, Campus de Teatinos s/n, 29071 Málaga, Spain. E-mail: carmenrd@uma.es

b Department of Chemistry "Giacomo Ciamician", University of Bologna, 40126 Bologna, Italy. Email: fabrizia.negri@unibo.it

c Department of Physical Chemistry, University of Alicante, 03080 Alicante, Spain

d INSTM, UdR Bologna, 40126 Bologna, Italy.

Table of Contents:

1.Computational Methods S2

2. DFT Calculations S4

3. References S18

1. COMPUTATIONAL METHODS

DFT, TD-DFT and SF-TD-DFT calculations. The structural and electronic properties of ten ICz isomers were determined with density functional theory (DFT) calculations using B3LYP[1,2] functional together with the 6-31G(d,p)[3,4] basis set employing the Gaussian 16 package. [5] First, the most stable CS geometry (with 3N-6 all-real vibrational frequencies) was found, and then we impose a broken-symmetry solution operating an unrestricted (U) wavefunction in order to determinate the OS geometry. [6] For the study of relative energies between different electronic states in all the systems investigated (*i.e.*, the energy difference between the CS and OS states or the diradical character), the M06-2X[7] functional was complementarily employed to study the effect of the functional choice. Theoretical Raman spectra were calculated at restricted B3LYP/6-31G** level considering the optimized diradical singlet OS geometry.[8] The computed frequencies were scaled by a factor of 0.9654.

The diradical character has been determined using two different descriptors. (a) First, the diradical character y_0 (note that a CS wavefunction has y_0=0 and a pure diradical state has y_0=1) was calculated from the occupation numbers of the highest occupied natural orbital and the lowest unoccupied natural orbital using the spin-projected formalism. [9] This parameter is obtained by the following equation.

$$y_0 = 1 - \frac{2T_i}{1+T_i^2} \qquad \text{where} \qquad T_i = \frac{n_{HONO-i} - n_{LUNO+i}}{2}$$

Where $n_{\mathrm{HONO}-i,\ \mathrm{LUNO}+i}$ are the occupation numbers of the occupied and unoccupied natural orbitals (NO). The diradical character corresponds to *i*=0. We note that the y_0 values are markedly dependent on the functional used.

(b) Second, we have carried out an analysis based on the fractional occupation number weighted density (FOD) whose main feature is to provide a robust and cost-effective information on the localization of “hot” electrons (strongly correlated and chemically active) in a molecule.[10] These calculations were performed using the ORCA 4.1 program.[11] We used the FOD analysis for a quantitative description of the open-shell singlet biradical character of these ground-state organic molecules. The number of hot electrons, N^{FOD}, was calculated at the FT-TPSS[12]/def2-TZVP[13] level at a default electronic temperature (T_{el}) of 5000 K. The isocontour value of the ρ^{FOD} plots was fixed to 0.005 e·bohr^{-3} allowing a systematic comparison between all the systems.

The diradical stability of the compounds under study is theoretically explored by means of different physical parameters:

1) <u>The singlet-triplet energy gap ($\Delta E_{S\text{-}T}$)</u>: the spin correction for $\Delta E_{S\text{-}T}$ was computed by the equation

$$\Delta E_{S-T} = (E_{OS} - E_T)\frac{\langle S^2\rangle_T}{\langle S^2\rangle_T - \langle S^2\rangle_{OS}}$$

Where E_{OS} and E_T correspond to the energies of the OS singlet and triplet state, with spin contamination values $\langle S^2\rangle_T$ and $\langle S^2\rangle_{OS}$ of the triplet and OS singlet state, respectively.

2) The effective electron exchange interaction (J_{ab}): the broken-symmetry (BS) method considered that spin correction is trustworthy for estimating the J_{ab} values, which were determined by using the following equation.

$$J_{ab} = \frac{(E_{OS} - E_T)}{\langle S^2 \rangle_T - \langle S^2 \rangle_{OS}}$$

The excitation energies of low-lying excited states are determined with several DFT-based computational schemes, encompassing standard time dependent DFT (TD-DFT) based on a CS reference configuration, and other flavours of the TD approach either based on an unrestricted spin paired reference configuration (TD-UDFT), or on spin-flip (SF)-TD-DFT. [14] Double excitations can be recovered from TD-DFT calculations with the SF scheme. SF-TD-DFT treats ground- and excited-state electron correlation on the same footing, while also incorporating some doubly excited configurations that are important for biradicals. Accordingly, this approach was employed to investigate the excitation energy of the low-lying H,H-L,L state of the molecules investigated. The SF-TD-DFT calculations were carried out in the collinear approximation as implemented in the Gamess 2016 package.[15]

NICS-XY scans were computed using the Aroma package[16-19] at the GIAO-B3LYP/6-311+G* level. NICS-XY values were taken 1.7 Å above the molecular plane and employed the σ-only model to take only π-contributions into consideration. The ACID plots were carried out by the CSGT method implemented in the Gaussian program (Keywords NMR=CSGT and IOp(10/93=1)) at B3LYP/6-311+G* level of theory. [20] The magnetic field has been oriented to be orthogonal to the ring plane and the chosen isosurface value has been 0.03.

CASSCF/NEVPT2 calculations. The singlet/triplet energy difference was estimated at the previously optimized OS UB3LYP/6-31G** geometries. State averaged (SA) complete active-space self-consistent-field (CASSCF) calculations were carried out using the same weight for the lowest singlet and triplet states. The active space comprised 10 electrons in 10 π orbitals (10,10) and was followed by second order perturbation theory NEVPT2[21] calculations to include dynamical electron correlation. All the CASSCF and NEVPT2 calculations were performed using the ORCA 5.0.1 program[22] using the def2-SVP basis set. The resolution of identity approximation and the related basis sets for both Coulomb and HF exchange integrals were used (RI-JK).[23]

2. DFT CALCULATIONS

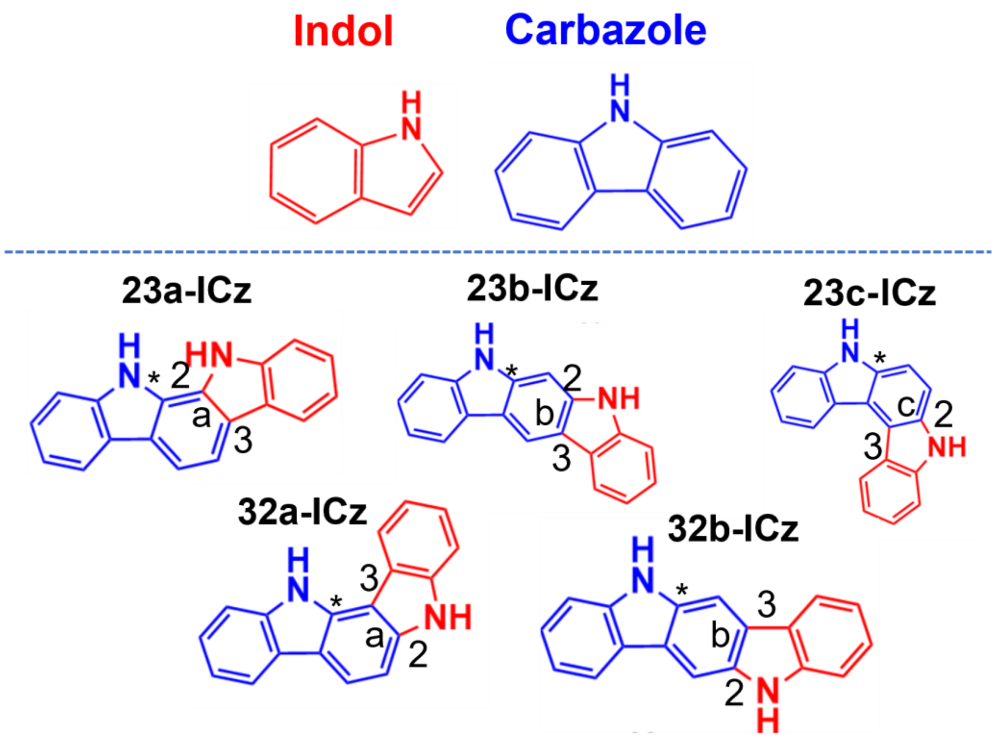

Figure S1. The five positional ICz isomers.

Table S1. Diradical indices y_0 and physical properties for ICz-based systems at UM06-2X/6-31G** level.

	$<S^2>_{OS}$	$<S^2>_T$	y_0 (PUM06-2X)	J_{ab} (kcal·mol^{-1})
***p*-23a-ICz**	1.02	2.05	0.65	-1.08
***p*-23b-ICz**	1.04	2.05	0.81	-0.01
***p*-23c-ICz**	1.04	2.05	0.79	-0.33
***p*-32a-ICz**	1.04	2.05	0.93	0.36
***p*-32b-ICz**	1.02	2.04	0.61	-1.59
***m*-23a-ICz**	1.05	2.04	0.97	0.02
***m*-23b-ICz**	1.04	2.05	0.87	-0.04
***m*-23c-ICz**	1.05	2.05	0.99	0.02
***m*-32a-ICz**	1.04	2.00	0.99	0.13
***m*-32b-ICz**	1.04	2.04	0.85	-0.18

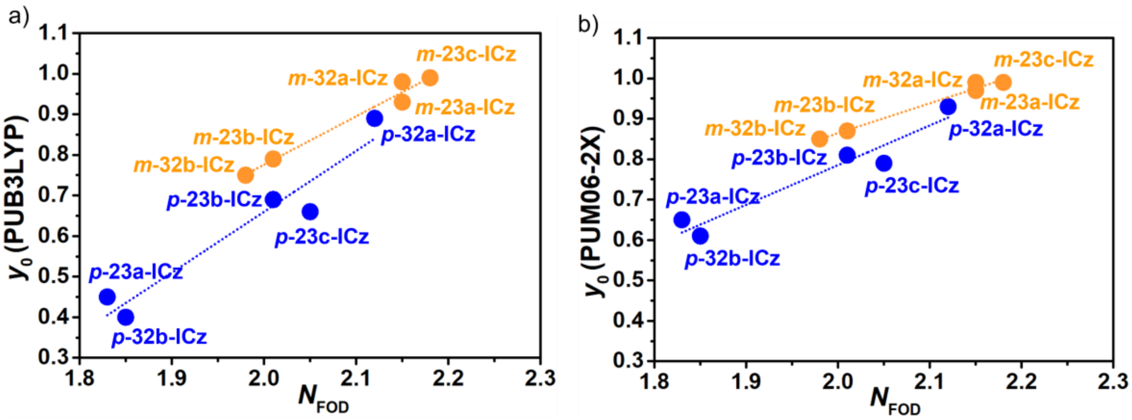

Figure S2. Comparison of diradical character (y_0) and the N^{FOD} values at UB3LYP (a) and UM06-2X (b) level of theory.

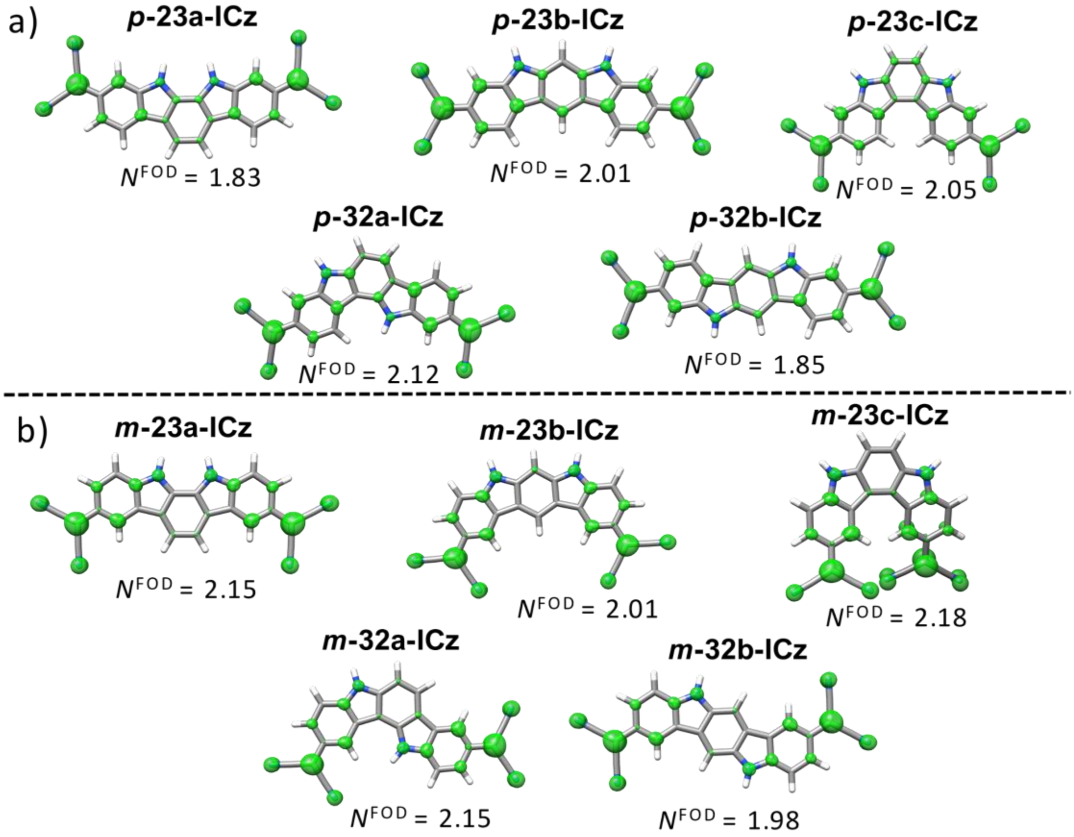

Figure S3. Isocontour plot of the FOD density (σ =0.005 e· bohr^{-3}) and predicted N^{FOD} values of the DCM para-substituted (a) and meta-substituted (b) ICz-based isomers.

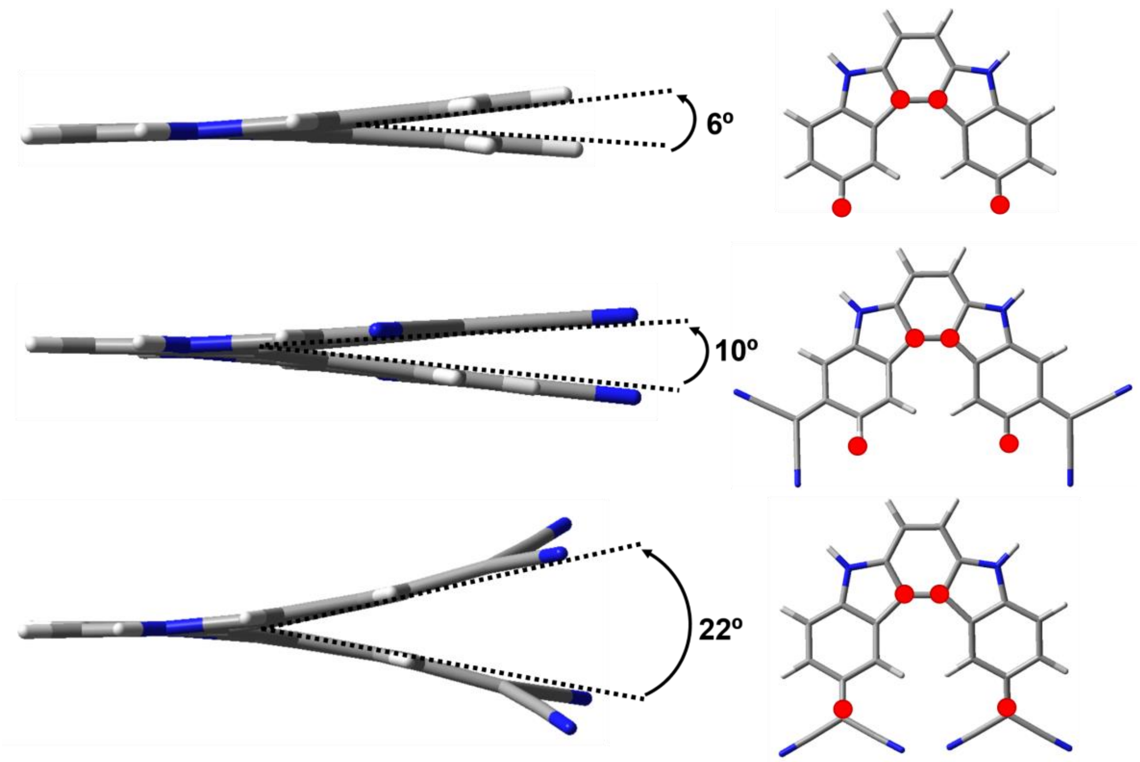

Figure S4. Top (right) and side (left) views of the optimized geometries for **23c-ICz** (top), ***p*-23c-ICz** (middle) and ***m*-23c-ICz** (bottom) calculated at the B3LYP/6-31G** level of theory. The bay dihedral values (°) are also shown.

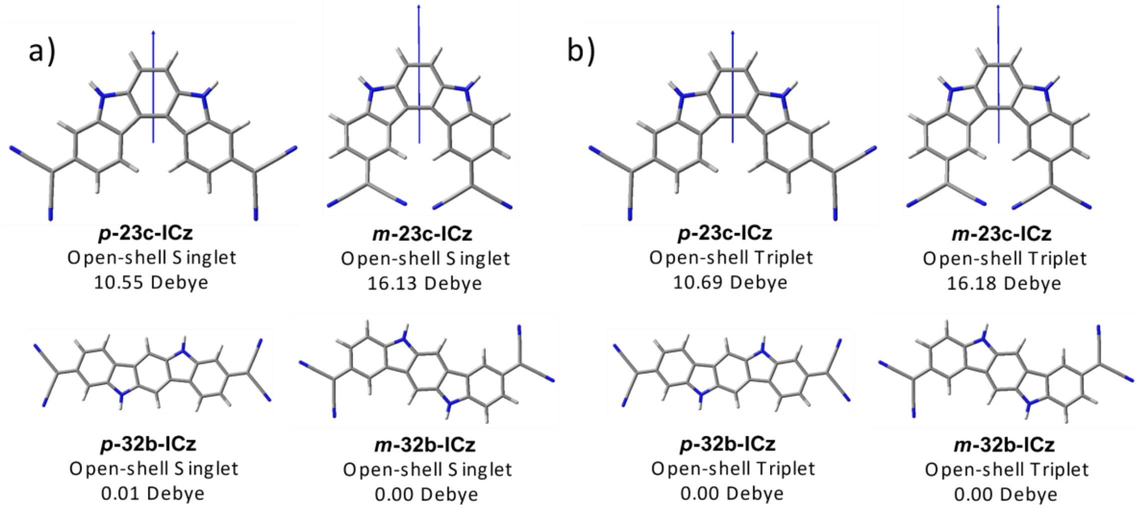

Figure S5. DFT-calculated (UB3LYP/6-31G**) dipole moments for ***p*-23c-ICz**, ***m*-23c-ICz**, ***p*-32b-ICz** and ***m*-32b-ICz** in their OS singlet (a) and triplet (b) states.

Table S2. Diradical indices y_0 and dipole moments for all ICz-based systems in their OS singlet and triplet states at UB3LYP/6-31G** level**.**

	y_0 (PUB3LYP)	Dipole moment (Debye)	
		OS	T
***p*-23a-ICz**	0.45	3.17	2.83
***p*-23b-ICz**	0.69	4.26	4.16
***p*-23c-ICz**	0.66	10.55	10.69
***p*-32a-ICz**	0.89	7.45	7.46
***p*-32b-ICz**	0.40	0.01	0.00
***m*-23a-ICz**	0.93	9.45	9.48
***m*-23b-ICz**	0.79	15.21	15.11
***m*-23c-ICz**	0.99	16.13	16.18
***m*-32a-ICz**	0.98	8.47	8.48
***m*-32b-ICz**	0.75	0.00	0.00

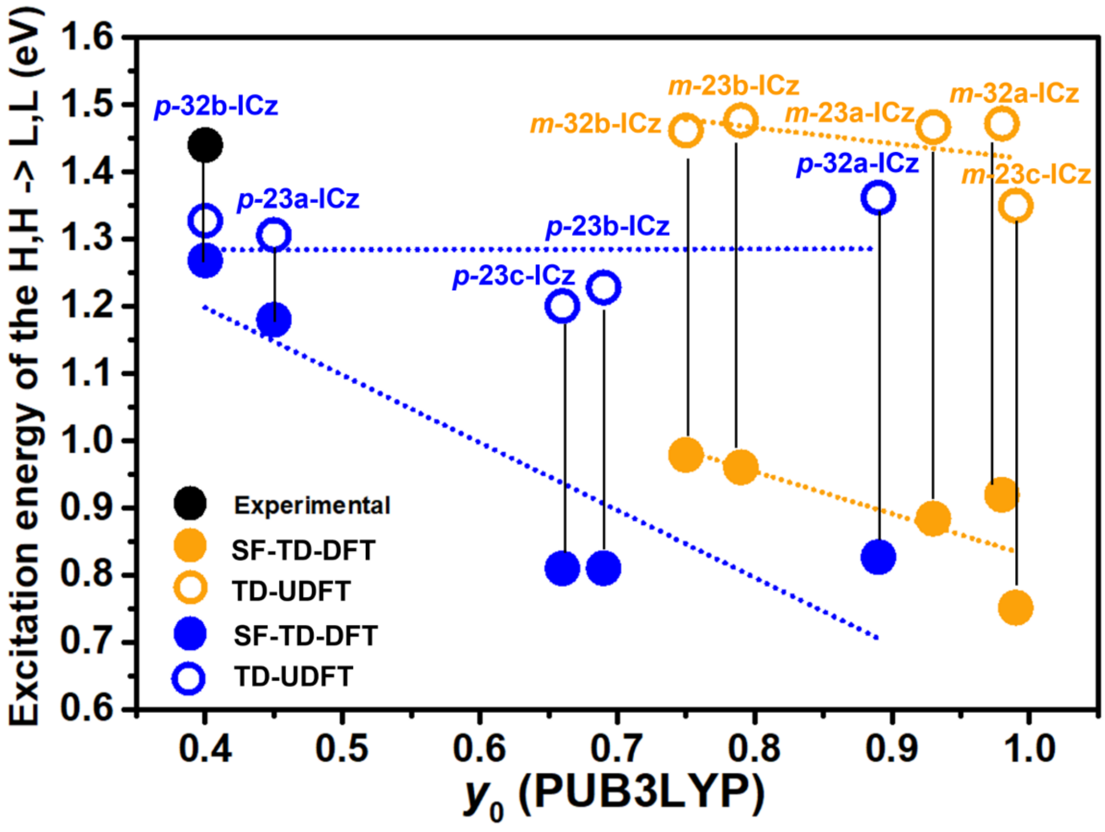

Figure S6. Calculated excitation energy of the one-photon forbidden (H,H→ L,L) state versus the computed projected diradical character at UB3LYP/6-31G** level of theory. Vertical bars indicate the compound to which computed data correspond.

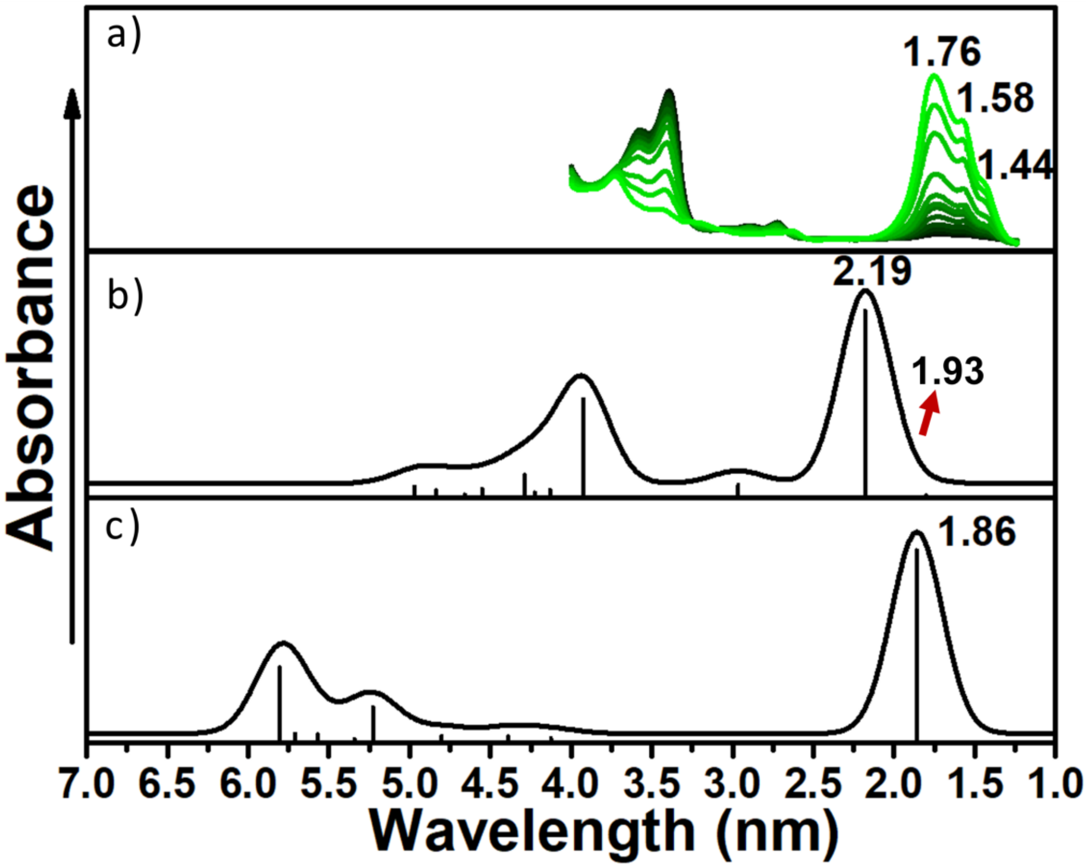

Figure S7. (a) UV/Vis-NIR absorption spectra of **p-32b-ICz** compound in o-dichlorobenzene upon heating from 300 to 410 K. (b) Simulated electronic absorption spectra at UM06-2X/6-31G** level of theory for OS structure. The red arrow shows the theoretical value of the one-photon forbidden (H,H → L,L) excited state. (c) Simulated electronic absorption spectra at M06-2X/6-31G** for CS structure.

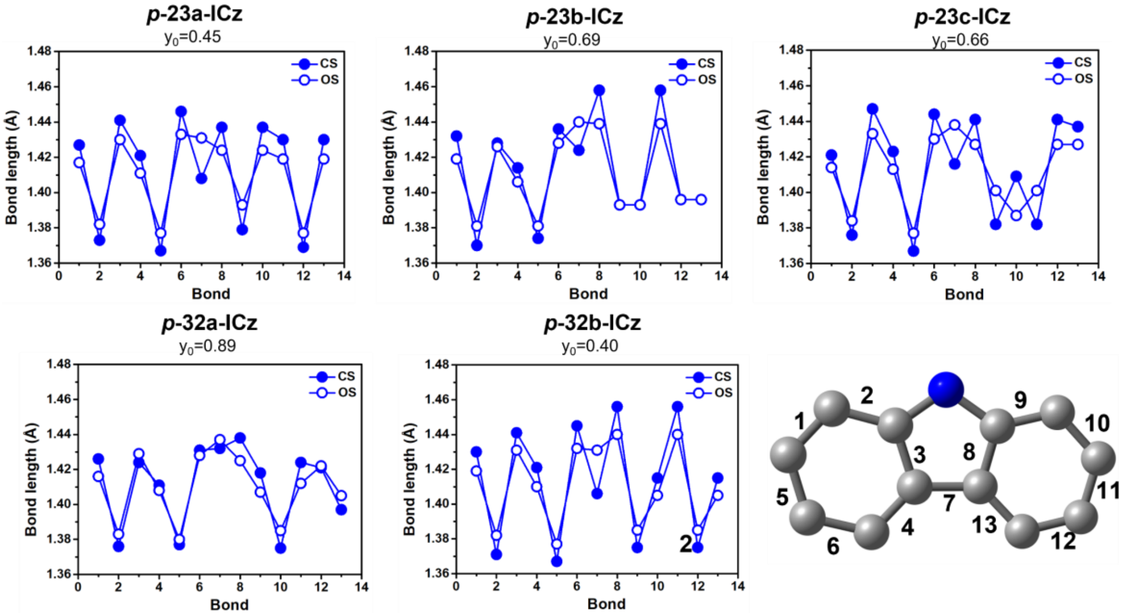

Figure S8. B3LYP/6-31G** computed bond-lengths (Å) for the optimized OS (empty circles) and CS (full circles) structures of para-DCM substituted compounds. The difference between CS and OS structures is more marked compared to meta-DCM substituted compounds shown in the next figure.

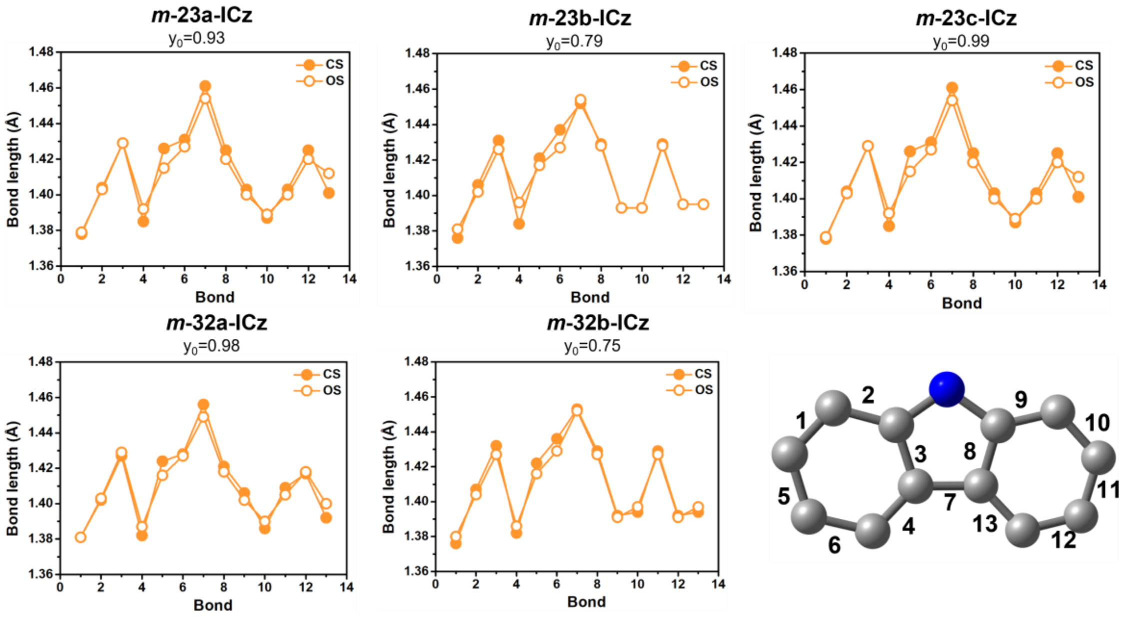

Figure S9. B3LYP/6-31G** computed bond-lengths (Å) for the optimized OS (empty circles) and CS (full circles) structures of meta-DCM substituted compounds. The difference between CS and OS structures is less marked compared to para-DCM substituted compounds shown in the previous figure.

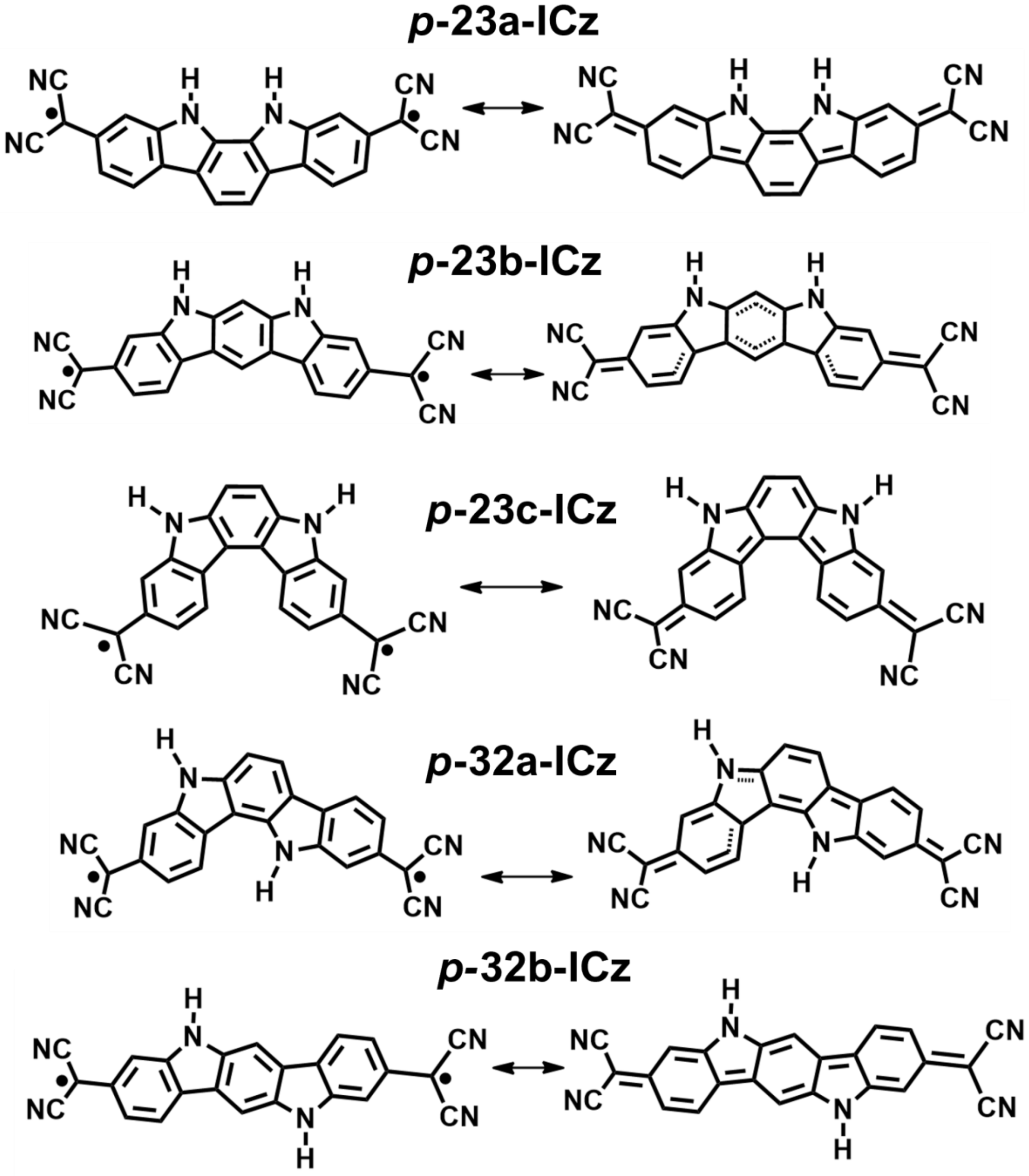

Figure S10. Equilibrium between a diradical character OS state (left) and a quinoid CS structure (right) for para-substituted isomers.

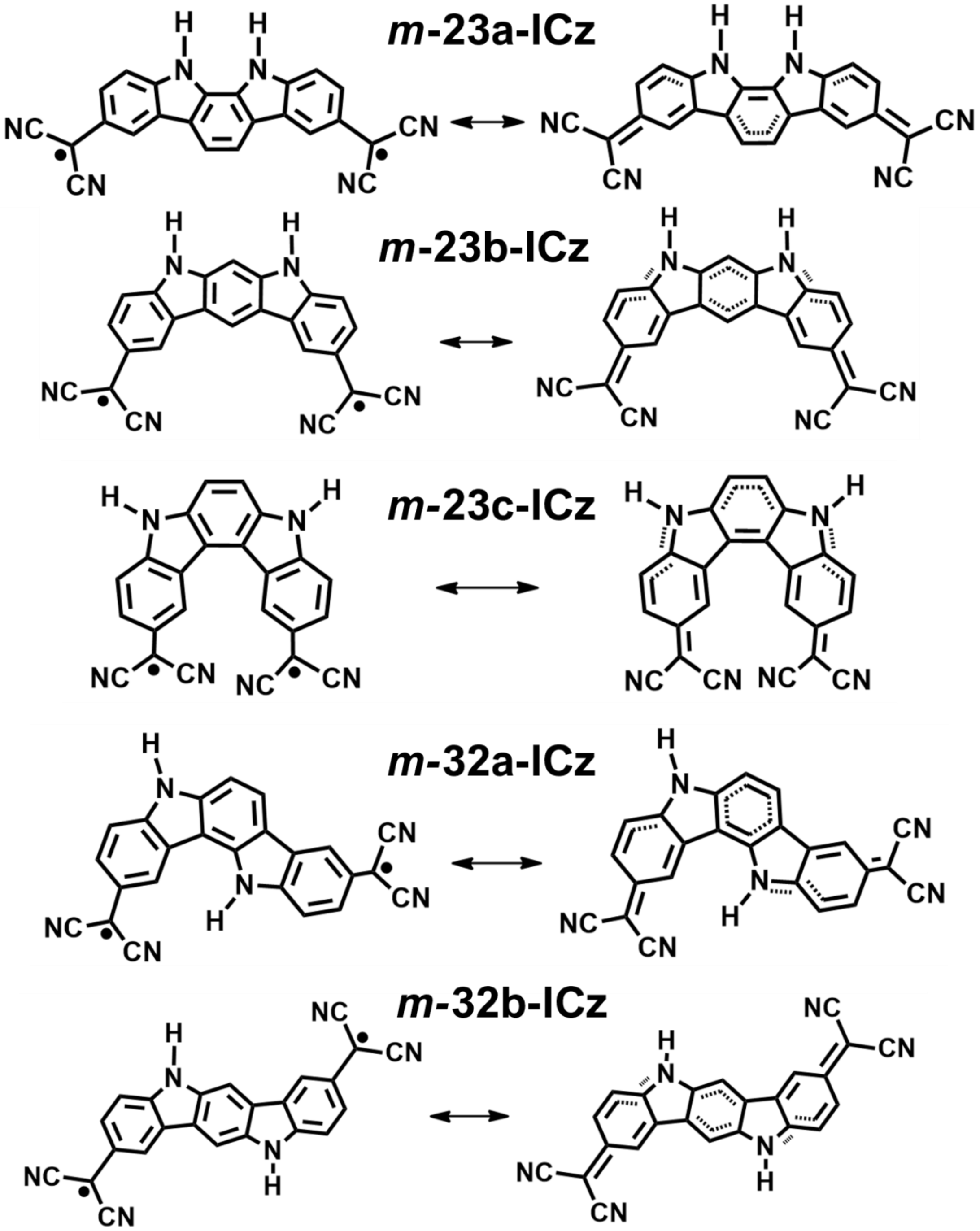

Figure S11. Equilibrium between a diradical character OS state (left) and a quinoid CS structure (right) for meta-substituted isomers.

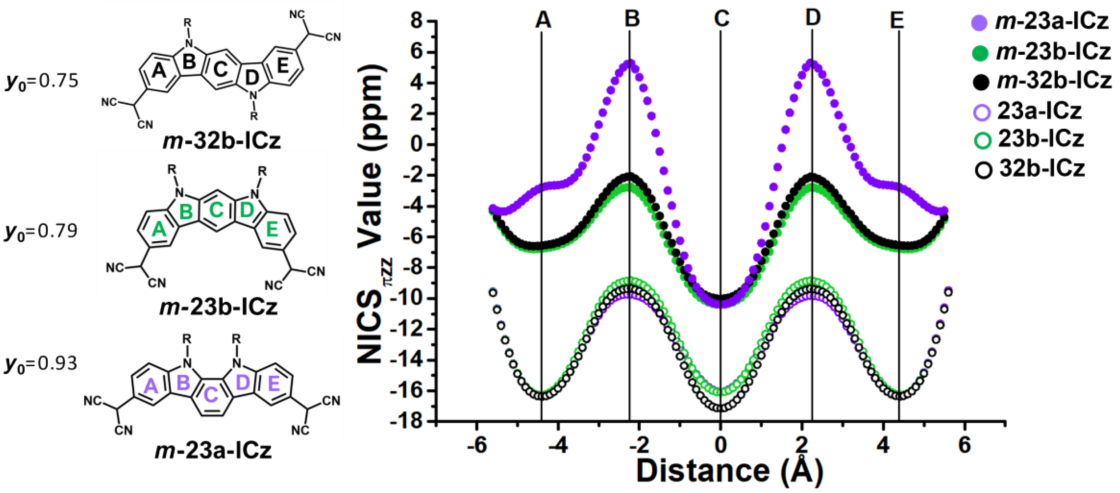

Figure S12. $NICS_{\pi zz}$-XY scan values (B3LYP/6-311+G* level) and chemical structures of **m-32b-ICz** (black circle), **m-23b-ICz** (green circle) and **m-23a-ICz** (purple circle). The $NICS_{\pi zz}$-XY scan values of unsubstituted isomer analogues (**32b-ICz**, **23b-ICz** and **23a-ICz**) are also shown for comparison.

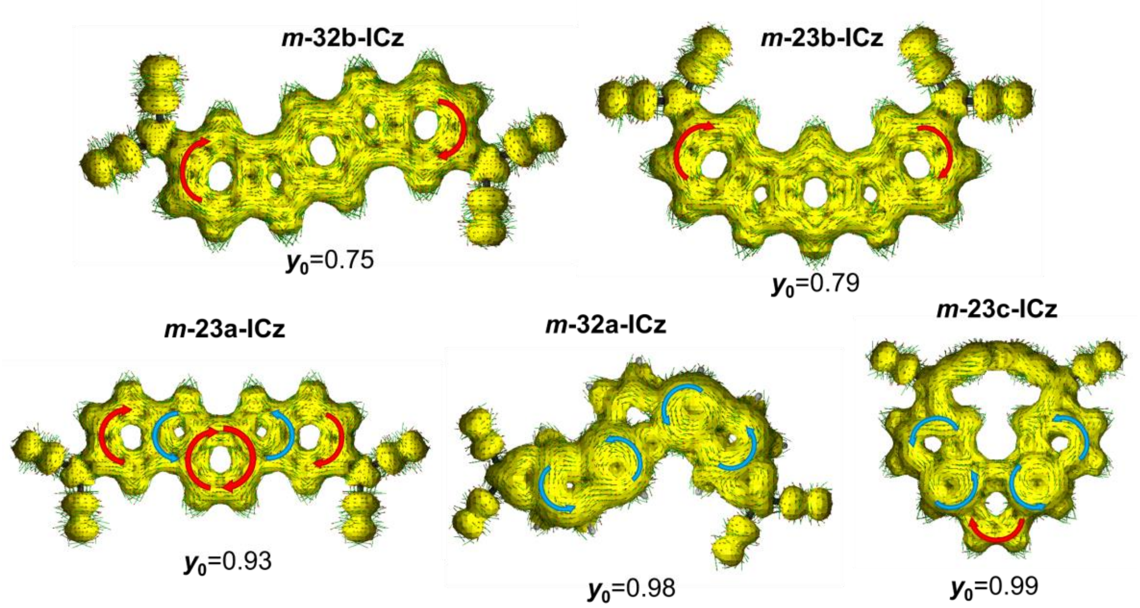

Figure S13. ACID plots of the induced ring current (B3LYP/6-311+G*) at an isosurface value of 0.03 of the meta-substituted ICz under study. The red arrows represent a diatropic (clockwise) ring current and the blue arrows correspond with a paratropic (counter-clockwise) ring current. The computed projected diradical character at UB3LYP/6-31G** level is also shown.

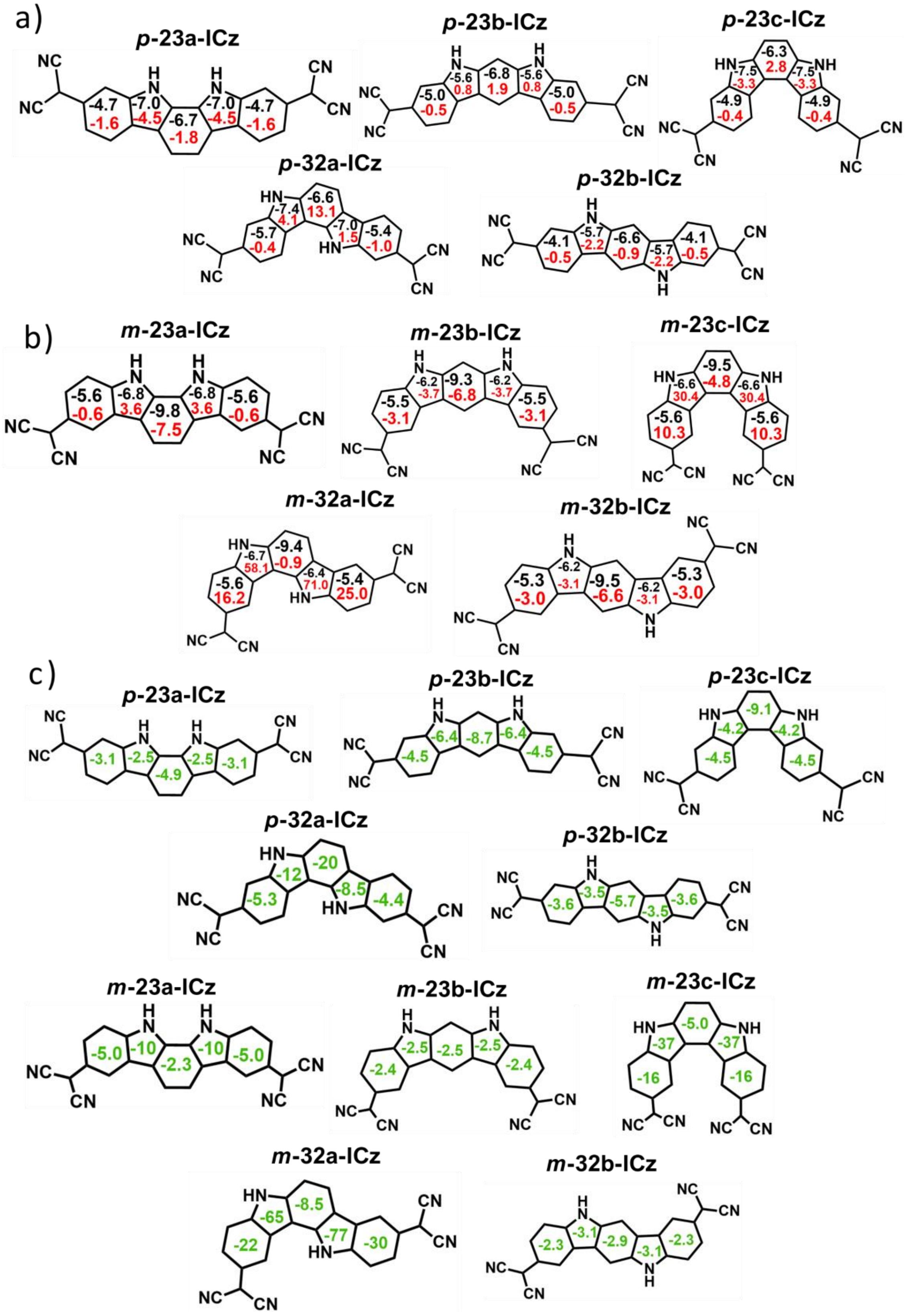

Figure S14. The NICS(0) values computed at UB3LYP/6-311+G* level for (a) para-substituted and (b) meta-substituted isomers at CS state (red values) and OS state (black values). (c) The differences between the NICS(0) values when going from CS to OS state for all ICz-based isomers.

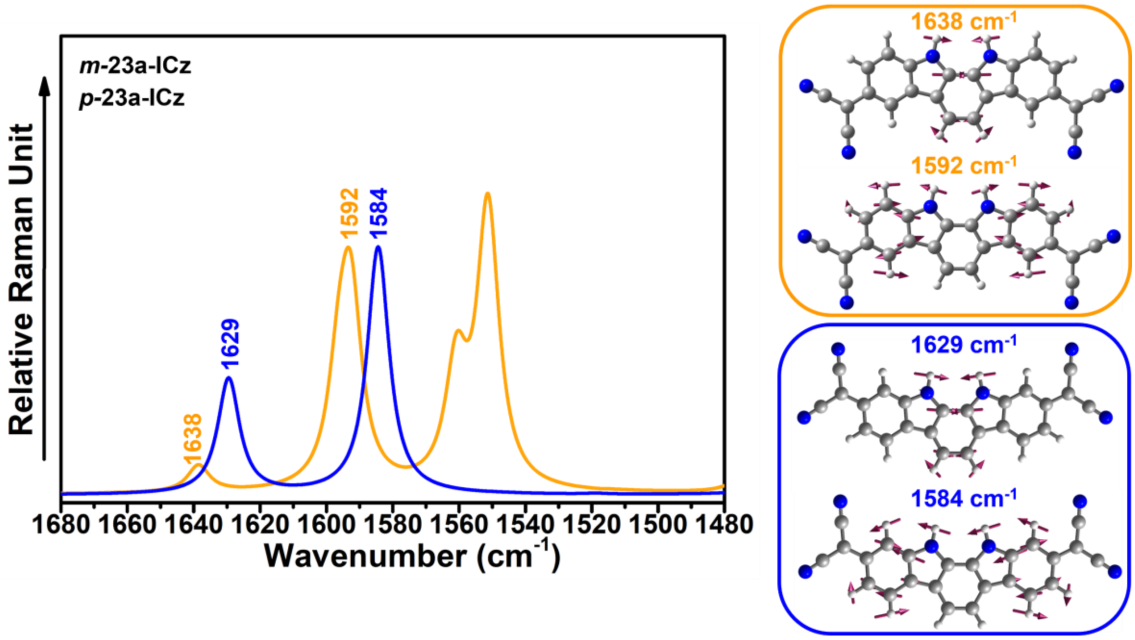

Figure S15. Left: Theoretical Raman spectra of ***m*-23a-ICz** (orange line) and ***p*-23a-ICz** (blue line) evaluated at B3LYP/6-31G** level considering the optimized diradical singlet OS geometry (f=0.9654). Right: Eigenvectors associated to the Raman bands ascribed to the CC stretching vibration of the ICz unit. The theoretical wavenumbers are also shown.

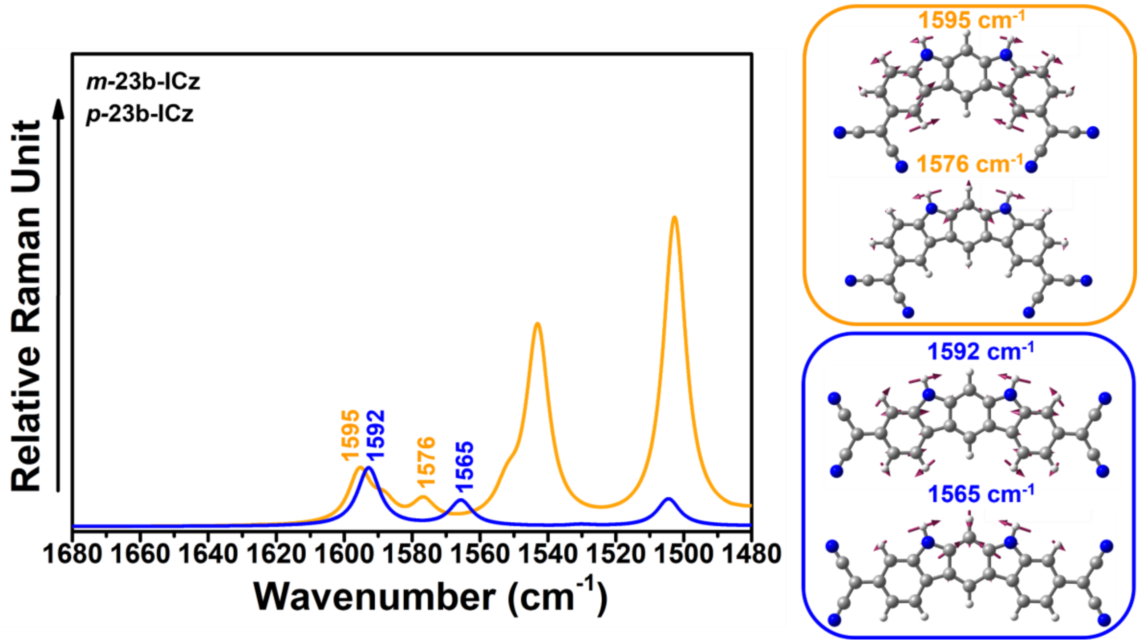

Figure S16. Left: Theoretical Raman spectra of *m*-**23b-ICz** (orange line) and ***p*-23b-ICz** (blue line) evaluated at B3LYP/6-31G** level considering the optimized diradical singlet OS geometry (f=0.9654). Right: Eigenvectors associated to the Raman bands ascribed to the CC stretching vibration of the ICz unit. The theoretical wavenumbers are also shown.

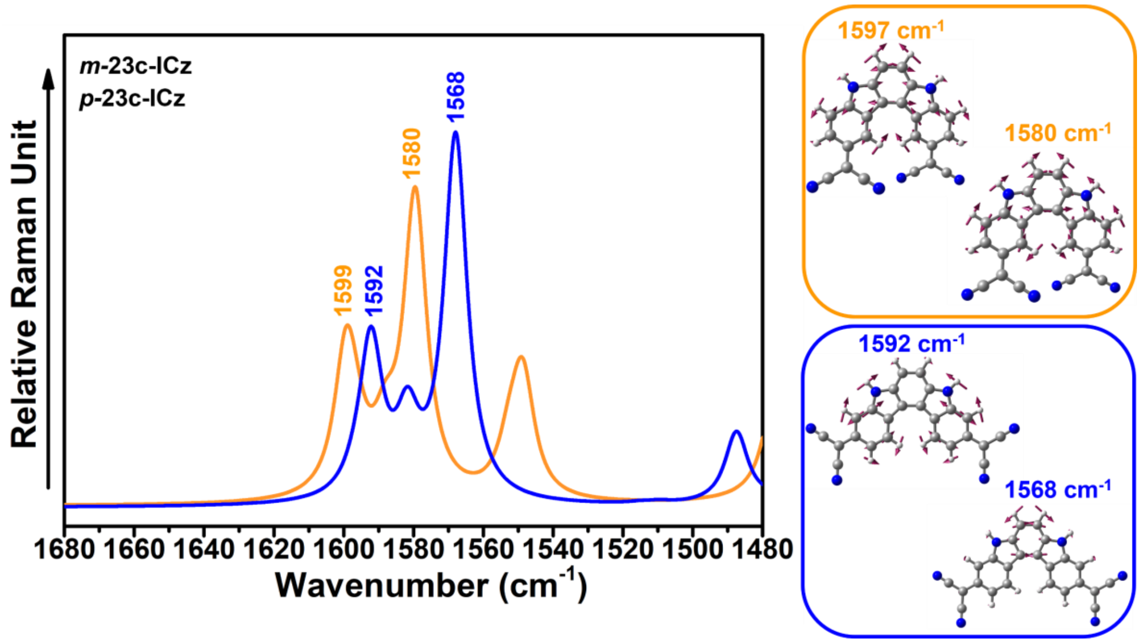

Figure S17. Left: Theoretical Raman spectra of ***m*-23c-ICz** (orange line) and ***p*-23c-ICz** (blue line) evaluated at B3LYP/6-31G** level considering the optimized diradical singlet OS geometry (f=0.9654). Right: Eigenvectors associated to the Raman bands ascribed to the CC stretching vibration of the ICz unit. The theoretical wavenumbers are also shown.

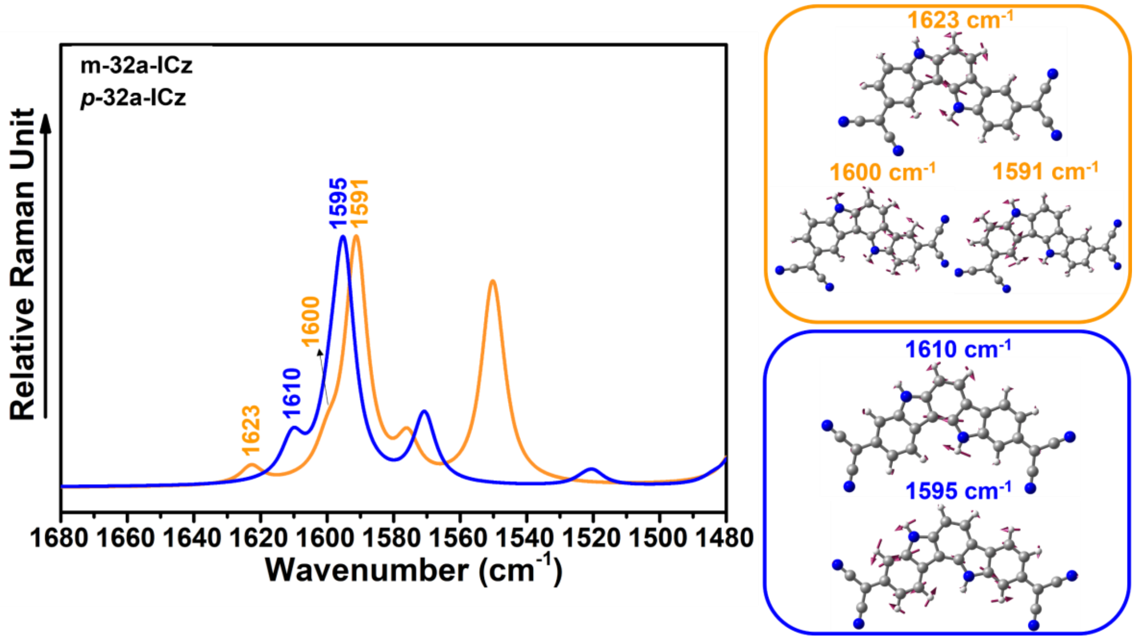

Figure S18. Left: Theoretical Raman spectra of ***m*-32a-ICz** (orange line) and ***p*-32a-ICz** (blue line) evaluated at B3LYP/6-31G** level considering the optimized diradical singlet OS geometry (f=0.9654). Right: Eigenvectors associated to the Raman bands ascribed to the CC stretching vibration of the ICz unit. The theoretical wavenumbers are also shown.

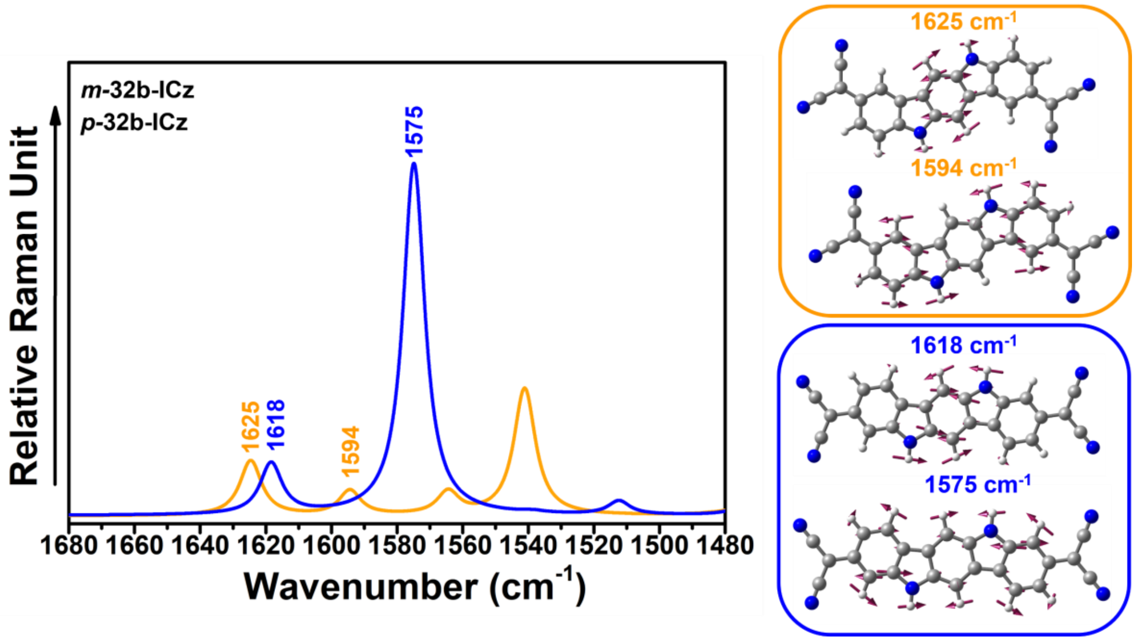

Figure S19. Left: Theoretical Raman spectra of ***m*-32b-ICz** (orange line) and ***p*-32b-ICz** (blue line) evaluated at B3LYP/6-31G** level considering the optimized diradical singlet OS geometry (f=0.9654). Right: Eigenvectors associated to the Raman bands ascribed to the CC stretching vibration of the ICz unit. The theoretical wavenumbers are also shown.

3. REFERENCES

1. Vosko, S. H.; Wilk, L.; Nusair, M., Accurate spin-dependent electron liquid correlation energies for local spin density calculations: a critical analysis. *Canadian Journal of Physics* **1980,** *58*, (8), 1200-1211.

2. Stephens, P. J.; Devlin, F. J.; Chabalowski, C. F.; Frisch, M. J., Ab Initio Calculation of Vibrational Absorption and Circular Dichroism Spectra Using Density Functional Force Fields. *The Journal of Physical Chemistry* **1994,** *98*, (45), 11623-11627.

3. Hehre, W. J.; Ditchfield, R.; Pople, J. A., Self—Consistent Molecular Orbital Methods. XII. Further Extensions of Gaussian—Type Basis Sets for Use in Molecular Orbital Studies of Organic Molecules. *The Journal of Chemical Physics* **1972,** *56*, (5), 2257-2261.

4. Francl, M. M.; Pietro, W. J.; Hehre, W. J.; Binkley, J. S.; Gordon, M. S.; DeFrees, D. J.; Pople, J. A., Self-consistent molecular orbital methods. XXIII. A polarization-type basis set for second-row elements. *The Journal of Chemical Physics* **1982,** *77*, (7), 3654-3665.

5. Frisch, M. J.; Trucks, G. W.; Schlegel, H. B.; Scuseria, G. E.; Robb, M. A.; Cheeseman, J. R.; Scalmani, G.; Barone, V.; Petersson, G. A.; Nakatsuji, H.; Li, X.; Caricato, M.; Marenich, A. V.; Bloino, J.; Janesko, B. G.; Gomperts, R.; Mennucci, B.; Hratchian, H. P.; Ortiz, J. V.; Izmaylov, A. F.; Sonnenberg, J. L.; Williams; Ding, F.; Lipparini, F.; Egidi, F.; Goings, J.; Peng, B.; Petrone, A.; Henderson, T.; Ranasinghe, D.; Zakrzewski, V. G.; Gao, J.; Rega, N.; Zheng, G.; Liang, W.; Hada, M.; Ehara, M.; Toyota, K.; Fukuda, R.; Hasegawa, J.; Ishida, M.; Nakajima, T.; Honda, Y.; Kitao, O.; Nakai, H.; Vreven, T.; Throssell, K.; Montgomery Jr., J. A.; Peralta, J. E.; Ogliaro, F.; Bearpark, M. J.; Heyd, J. J.; Brothers, E. N.; Kudin, K. N.; Staroverov, V. N.; Keith, T. A.; Kobayashi, R.; Normand, J.; Raghavachari, K.; Rendell, A. P.; Burant, J. C.; Iyengar, S. S.; Tomasi, J.; Cossi, M.; Millam, J. M.; Klene, M.; Adamo, C.; Cammi, R.; Ochterski, J. W.; Martin, R. L.; Morokuma, K.; Farkas, O.; Foresman, J. B.; Fox, D. J. Gaussian 16 Rev. A.03, *Wallingford, CT*, **2016**.

6. Lahti, P. M.; Ichimura, A. S.; Sanborn, J. A., Methodologies for Computational Studies of Quininoidal Diiminediyls: Biradical vs Dinitrene Behavior. *The Journal of Physical Chemistry A* **2001,** *105*, (1), 251-260.

7. Zhao, Y.; Truhlar, D. G., Density Functionals with Broad Applicability in Chemistry. *Accounts of Chemical Research* **2008,** *41*, (2), 157-167.

8. Fazzi, D.; Canesi, E. V.; Negri, F.; Bertarelli, C.; Castiglioni, C., Biradicaloid Character of Thiophene-Based Heterophenoquinones: The Role of Electron–Phonon Coupling. *ChemPhysChem* **2010,** *11*, (17), 3685-3695.

9. Yamaguchi, K., The electronic structures of biradicals in the unrestricted Hartree-Fock approximation. *Chemical Physics Letters* **1975,** *33*, (2), 330-335.

10. Pérez-Guardiola, A.; Sandoval-Salinas, M. E.; Casanova, D.; San-Fabián, E.; Pérez-Jiménez, A. J.; Sancho-García, J. C., The role of topology in organic molecules: origin and comparison of the radical character in linear and cyclic oligoacenes and related oligomers. *Physical Chemistry Chemical Physics* **2018,** *20*, (10), 7112-7124.

11. Neese, F., The ORCA program system. *Wiley Interdisciplinary Reviews: Computational Molecular Science* **2012,** *2*, (1), 73-78.

12. Tao, J.; Perdew, J. P.; Staroverov, V. N.; Scuseria, G. E., Climbing the Density Functional Ladder: Nonempirical Meta--Generalized Gradient Approximation Designed for Molecules and Solids. *Physical Review Letters* **2003,** *91*, (14), 146401.

13. Weigend, F.; Ahlrichs, R., Balanced basis sets of split valence, triple zeta valence and quadruple zeta valence quality for H to Rn: Design and assessment of accuracy. *Physical Chemistry Chemical Physics* **2005,** *7*, (18), 3297-3305.

14. Shao, Y.; Head-Gordon, M.; Krylov, A. I., The spin–flip approach within time-dependent density functional theory: Theory and applications to diradicals. *The Journal of Chemical Physics* **2003,** *118*, (11), 4807-4818.
15. Schmidt, M. W.; Baldridge, K. K.; Boatz, J. A.; Elbert, S. T.; Gordon, M. S.; Jensen, J. H.; Koseki, S.; Matsunaga, N.; Nguyen, K. A.; Su, S.; Windus, T. L.; Dupuis, M.; Montgomery Jr, J. A., General atomic and molecular electronic structure system. *Journal of Computational Chemistry* **1993,** *14*, (11), 1347-1363.
16. Rahalkar, A. S., A., https://chemistry.technion.ac.il/en/team/amnon-stanger/.
17. Stanger, A., Nucleus-Independent Chemical Shifts (NICS): Distance Dependence and Revised Criteria for Aromaticity and Antiaromaticity. *The Journal of Organic Chemistry* **2006,** *71*, (3), 883-893.
18. Stanger, A., Obtaining Relative Induced Ring Currents Quantitatively from NICS. *The Journal of Organic Chemistry* **2010,** *75*, (7), 2281-2288.
19. Gershoni-Poranne, R.; Stanger, A., The NICS-XY-Scan: Identification of Local and Global Ring Currents in Multi-Ring Systems. *Chemistry – A European Journal* **2014,** *20*, (19), 5673-5688.
20. Herges, R.; Geuenich, D., Delocalization of Electrons in Molecules. *The Journal of Physical Chemistry A* **2001,** *105*, (13), 3214-3220.
21. Angeli, C., Cimiraglia, R., Evangelisti, S., Leininger, T., and Malrieu, J. P. Introduction of n-electron valence states for multireference perturbation theory. *The Journal of Chemical Physics*, **2001**, *114*, (23), 10252-10264
22. Neese, F., Wennmohs, F., Becker, U., & Riplinger, C. The ORCA quantum chemistry program package. *The Journal of chemical physics*, **2020**, *152*, (22), 224108.
23. Weigend, F. Hartree–Fock exchange fitting basis sets for H to Rn. *Journal of computational chemistry*, **2008**, *29*, (2), 167-175.
